# SUMOylation of OsPSTOL1 is essential for regulating phosphate starvation responses in rice and *Arabidopsis*

**DOI:** 10.3389/fpls.2024.1274610

**Published:** 2024-03-07

**Authors:** Vaishnavi Mukkawar, Dipan Roy, Kawinnat Sue-ob, Andrew Jones, Cunjin Zhang, Prakash Kumar Bhagat, Sumesh M. Kakkunnath, Sigrid Heuer, Ari Sadanandom

**Affiliations:** ^1^ Department of Biosciences, Durham University, Durham, United Kingdom; ^2^ Department of Biochemistry, Cell and Systems Biology, Institute of System, Molecular and Integrative Biology, University of Liverpool, Liverpool, United Kingdom; ^3^ Department of Crop Science, Cambridge Discovery LTD, Cambridge, United Kingdom

**Keywords:** post-translational modification, SUMOylation, phosphate-starvation tolerance 1 (OsPSTOL1), inorganic phosphate, phosphate deficiency

## Abstract

Although rice is one of the main sources of calories for most of the world, nearly 60% of rice is grown in soils that are low in phosphorus especially in Asia and Africa. Given the limitations of bioavailable inorganic phosphate (Pi) in soils, it is important to develop crops tolerant to low phosphate in order to boost food security. Due to the immobile nature of Pi, plants have developed complex molecular signalling pathways that allow them to discern changes in Pi concentrations in the environment and adapt their growth and development. Recently, in rice, it was shown that a specific serine–threonine kinase known as *Phosphorus-starvation tolerance 1 (PSTOL1)* is important for conferring low phosphate tolerance in rice. Nonetheless, knowledge about the mechanism underpinning PSTOL1 activity in conferring low Pi tolerance is very limited in rice. Post-translation modifications (PTMs) play an important role in plants in providing a conduit to detect changes in the environment and influence molecular signalling pathways to adapt growth and development. In recent years, the PTM SUMOylation has been shown to be critical for plant growth and development. It is known that plants experience hyperSUMOylation of target proteins during phosphate starvation. Here, we demonstrate that PSTOL1 is SUMOylated *in planta*, and this affects its phosphorylation activity. Furthermore, we also provide new evidence for the role of SUMOylation in regulating PSTOL1 activity in plant responses to Pi starvation in rice and *Arabidopsis*. Our data indicated that overexpression of the non-SUMOylatable version of OsPSTOL1 negatively impacts total root length and total root surface area of rice grown under low Pi. Interestingly, our data also showed that overexpression of OsPSTOL1 in a non-cereal species, *Arabidopsis*, also positively impacts overall plant growth under low Pi by modulating root development. Taken together our data provide new evidence for the role of PSTOL1 SUMOylation in mediating enhanced root development for tolerating phosphate-limiting conditions.

## Introduction

Abiotic stresses such as nutrient deficiency in soil is a major threat to food security. The three most important macronutrients are nitrogen, potassium, and phosphorus, which are required for plant growth and development. However, the importance of phosphorus is receiving widespread attention because it is an essential nutrient that comes from a non-renewable resource, thereby making phosphorus a finite resource (Lott et al., 2011). Phosphorus (P) is an indispensable element for all living organisms, as it is the main component of biomolecules—such as nucleic acids and lipids (Carlos Barragán-Rosillo et al., 2021). Plants acquire inorganic phosphate (orthophosphate; Pi) from the soil, but the accessibility of phosphorus is limited because of the high tendency of Pi to be complexed with metal ions such as iron, aluminum, or calcium. As a result, Pi fertilisers are rendered very ineffective because only 10%–25% of Pi from fertilisers is taken up by plants and the rest of the Pi is bound to iron or aluminium in the soil (Crombez et al., 2019). Phosphate nutrient limitation greatly affects crop yield in over 60% of the world’s fertile land. Therefore, a profound grasp of the regulatory mechanisms of Pi signalling and sensing in model crops and plants such as rice and *Arabidopsis*, respectively, will aid the development of Pi tolerant crops for better food security.

Plants being sessile organisms are constrained to their site of germination and have to integrate their growth and development with their vicinal environment. As a result, plants have evolved intricate mechanisms to maintain developmental plasticity for their entire life cycle (Qazi et al., 2019; Clark et al., 2022). One of these mechanisms, post-translational modifications (PTMs) is emerging as a critical process for maintaining developmental plasticity in plants but also regulating key developmental pathways in response to abiotic or biotic cues from the environment.

The role of post-translational modifications such as SUMOylation in the regulation of phosphate starvation responses in *Arabidopsis* and rice has been reported previously. Miura et al. (2005) demonstrated that SUMO E3 ligase (AtSIZ1) modulates root architecture under low Pi where *siz1* mutant displayed exaggerated Pi starvation responses. In Pi starvation regulatory network, *AtPHR1* transcription factor plays an important role known to be SUMOylated by AtSIZ1, and this is the only SUMO ligase in *Arabidopsis* demonstrated to be involved in phosphate stress responses (Miura et al., 2005). These studies clearly indicate an emerging role for SUMOylation in phosphate signalling in *Arabidopsis.* As way of translating the data from *Arabidopsis* to rice, OsSIZ1 was also characterised in rice during Pi starvation. The data suggest that OsSIZ1 can act both positively and negatively to regulate genes involved in Pi starvation responses in rice, which agrees with the literature in *Arabidopsis* (Wang et al., 2015). Additionally, the functional role of other SUMO components was also investigated. Mutations in *OsSAE1a* affected shoot and root length in high and low Pi when compared to WT plants. Further investigations suggested that there is a higher uptake of Pi in roots of OsSAE1a-silenced RNAi lines, indicating its negative role in Pi acquisition. In contrast, there was a similar distribution of Pi in shoot and roots between WT and *OsSAE1a* RNAi lines regardless of Pi supply, suggesting that OsSAE1a is not involved in the mobilisation of Pi (Pei et al., 2020). This evidence indicates an important role for post-translational modifications, in particular, SUMOylation in Pi starvation response in plants.

In recent years, alternate approaches involving the identification of new molecular markers that are of particular value for the development of phosphorus-efficient rice varieties have been implemented. In a forward genetic screen to identify rice varieties with enhanced phosphate starvation tolerance, *Phosphate uptake 1* (*Pup1*) locus on chromosome 12 from Kasalath, an aus-type rice variety, was identified and sequenced. *Pup1* near-isogenic lines (NILs) were shown to have improved phosphorus uptake efficiency compared to Nipponbare (Neelam et al., 2017) when grown in phosphorus-deficient soil. Subsequently, the *Pup1* locus in Kasalath was sequenced, and it showed the presence of a nearly 90-kb transposon-rich insertion–deletion (INDEL) region. When the sequence was aligned with the Nipponbare genome, the entire region was absent from the genome of Nipponbare (Heuer et al., 2009). Further investigation of *Pup1* revealed that *Pup1-*related serine–threonine kinase, known as phosphorus-starvation tolerance 1 (OsPSTOL1), was responsible for conferring rice plant tolerance to low phosphorus availability in soils. Overexpression of this gene in Nipponbare and IR64 boosted grain yield by 60% under Pi-deficient soil. Further analysis showed that PSTOL1 is a key regulator of root growth development in rice, which will improve the uptake of Pi from the soil (Gamuyao et al., 2012). Nonetheless, the knowledge of the OsPSTOL1-dependent mechanism regulating root architecture in low Pi is still very limited in rice. Here, we demonstrate that mutations in the SUMO sites of OsPSTOL1 affect the SUMOylation and autophosphorylation status of OsPSTOL1 kinase. Furthermore, we also showed that non-SUMOylatable version of OsPSTOL1 protein in rice and *Arabidopsis* is essential for regulating phosphate starvation responses by regulating root growth parameters, thereby, establishing the role of SUMOylation in regulating OsPSTOL1 activity in mediating low phosphate tolerance in rice and *Arabidopsis*.

## Materials and methods

### Plant treatment and plant growth analysis

Seeds of transformed rice and *Arabidopsis* plants (two independent lines from homozygous T3 generation) were used for the assessment of the response of the plants overexpressing OsPSTOL1 WT and non-SUMOylatable version of OsPSTOL1 (OsPSTOL1^2K/R^) to high and low Pi conditions.

The overexpressing OsPSTOL1 and OsPSTOL1^2K/R^ *Arabidopsis* transgenic plants were generated in *Col-0* background using floral dip method (Clough and Bent, 1998). *Arabidopsis* seeds were sterilised using chlorine gas generated from the reaction between 3 ml concentrated hydrochloric acid and 97 ml of 12% hypochlorite solution. The sterilisation was set up in a closed box in a fume hood. The seeds were allowed to sterilise for 10–12 h before being ventilated in laminar hood to remove chlorine gas. The seeds were placed on 1/2 Murashige and Skoog (MS) with phytoagar (Duchefa Biochemie, Catalog no. P1003), and the plates were sealed with a micropore tape (3M). The sealed plates were transferred for stratification at 4°C for 3 days. After 3 days, the plates were then placed vertically at 21°C temperature in 16/8-h light/dark programme in a Sanyo growth cabinet. Seedlings 4 days old (n = 50) were transferred to basal medium containing full-strength Murashige and Skoog (MS) media with 1.25 mM and 3 μM of KH_2_PO_4_ (Merck, Catalog no. P0662-25G) as high and low Pi, respectively. pH was maintained at 5.7. The plant response to high and low Pi was analysed until day 11 post-transferring. The data were representative of three biological replicates, each with 16–18 seedlings.

Mature rice seeds were dehusked with a rice husker, and seeds were collected into a sterile 50-ml tube. The seeds were surface sterilised in 20 ml of 70% ethanol for 10 s and then in 25 ml of 2% sodium hypochlorite for 22–25 min with shaking. The seeds were rinsed with sterile water for five times. Extra water was removed, and the seeds were placed on 1/2 MS basal salt mixture (Duchefa Biochemie, Catalog no. M0222) plates with phytoagar (Hiei and Komari, 2008). The seeds were stratified at 28°C for 3 days before transferring them to light at 28°C, and the seeds were transferred to 24 h light for 1 day. Seeds (n = 16) with similar germination rates were transferred to Yoshida media, modified from the composition previously described by Gamuyao et al. (2012) for phosphate concentration—P-sufficient (100μM) and P-deficient (3μM)—and the phenotype was observed after 28 days of treatment. The data were representative of three biological replicates, each with 16 seedlings.

### Vector construction and plant transformation

Full-length coding sequence (CDS) of *OsPSTOL1* was amplified from Kasalath genomic DNA and cloned into pDTOPO vector. The gene was subcloned into binary vector pIPKb002, which has ubiquitin promoter. The construct was introduced into *Agrobacterium tumefaciens* strain EHA105 and then transformed into rice (*O. sativa* cv. Nipponbare) (Hiei and Komari, 2008).

Gene constructs (OsPSTOL1 WT and OsPSTOL1^2K/R^) were sub-cloned upstream of YFP in the gateway destination vector pEARLYGATE 104 using Gateway LR clonase. The vector has an enhanced CaMV 35S promoter to drive gene expression with an N-terminal YFP tag. These constructs were transformed into *Agrobacterium tumefaciens* strain GV3101.

### Site-directed mutagenesis

SUMO sites mutated version of *OsPSTOL1* were generated by site-directed mutagenesis using *OsPSTOL1 WT* pDTOPO clone as template. The introduction of mutation was then confirmed by sequencing (Verma et al., 2021).

### Selection of transgenic rice by evaluating the transgene copy number

At T0 stage, the pure genomic DNA from rice seedlings was isolated using the DNeasy^®^Plant Mini kit (Qiagen). SYBR Green qPCR (quantitative polymerase chain reaction) was carried out in thermal cycler Rotorgene Q. To calculate the PSTOL1 copy number, a relative quantification method was used. In this approach, the absolute value was used: one for the endogenous reference gene (sucrose phosphate synthase, SPS) and one for the target specific gene from transgenic plants (Hygromycin gene). Primers used for evaluating the transgene copy number are listed in **Supplementary Table S1**. The relative values are then compared to positive control, which, on previous investigation, had shown single-copy SPS gene in GM (genetically modified) rice (Xiujie et al., 2019). The quantitative RT-PCR result of the SPS gene amplification was to represent total rice genome copy number, which was designed to normalise the reaction, thereby enabling estimation of the transgene copy number.

### SDS-PAGE and Western blotting

Sodium dodecyl sulphate polyacrylamide gel electrophoresis (SDS-PAGE) was used to analyse and separate the proteins according to molecular weight. These gels can cast as follows: stacking gel, 5% acrylamide, 0.125 M Tris pH 6.8, 0.1% SDS, 0.1% ammonium persulphate and 0.01% TEMED; resolving gel, ranged from 10% to 15% acrylamide, 0.375M Tris pH 8.8, 0.1% SDS, 0.1% ammonium persulphate, and 0.04% TEMED. The gel (without stacking gel) was submersed in transfer buffer for equilibration. Meanwhile, a PVDF membrane (Merck, Catalog no. IPVH00010) was submersed in 100% methanol for 1 min. The membrane was then soaked in transfer buffer for 5 min. Blotting paper and sponges (Bio-Rad, Catalog no. 1703930) were prepared within a clamp ready blotting cassette. The gel and PVDF membrane were sandwiched between the blotting paper and sponges in the cassette. The proteins were allowed to transfer overnight at 25 V at 4°C from SDS-PAGE onto the membrane. The membrane was removed and was incubated in 5% semi-skimmed milk (blocking solution) for 2 h at room temperature. The membrane will be then incubated with primary antibody–anti-GFP antibody (dilution 1:5000; Abcam, Catalog no. ab6556) or anti-MBP monoclonal antibody (dilution 1:10,000; New England Biolabs, Catalog no. E8200) or anti-AtSUMO1/2 (dilution 1:2,500; generated against AtSUMO1/2 in rabbit), where the incubation times varied from 3 h at room temperature to overnight at 4°C depending on the antibody. After primary antibody incubation, the membrane was rinsed with 1× TBST for 5 min and then incubated with secondary horseradish peroxidase-conjugated antibodies (anti-rabbit for anti-GFP and anti-AtSUMO1/2 and anti-mouse for anti-MBP) for 1 h at room temperature. The ECL solution 1 and solution 2 (Bio-Rad, Catalog no. 1705060) were mixed in 1:1 ratio, and the membrane was incubated with ECL solution mix for 1 min and sealed in a light-proof cassette. In a dark room, the X-ray film was placed on the membrane and removed after various time periods. The reaction between ECL and HRP antibody caused light, and the exposed film was developed using a Xograph Compact 4x Automated Processor (Xograph Imaging Systems).

### Protein extraction from *N. benthamiana* leaves, rice, and *Arabidopsis* seedlings

35S::YFP-OsPSTOL1 WT and 35S::YFP-OsPSTOL1^2K/R^ constructs were transformed into *Agrobacterium* strain GV3101 and were coinfiltrated along with 35S::HA-SUMO and P19 into *N. benthamiana*. The infiltrated samples were collected after 3 days and frozen in liquid nitrogen. For protein extraction from rice and *Arabidopsis* seedlings, transgenic seeds of rice and *Arabidopsis* were placed on 1/2 MS plate and were grown 7–10 day. Samples were harvested and frozen in liquid nitrogen. Samples were ground into fine powder by liquid nitrogen in a mortar and pestle. PVPP and SUMO extraction buffer were added sequentially to the fine powder. The samples were then defrosted and centrifuged at 8,500 rpm for 15 min. The supernatant was transferred to a new microcentrifuge tube and centrifuged again at 14,000 rpm for 5 min, and the collected supernatant was incubated with MACS^®^ microbeads (Miltenyi Biotech) for 30 min. After incubation, in order to capture bound target protein, the supernatant was passed through a magnetic column (Miltenyi Biotech, Catalog no. 130-042-701), and the column was subsequently washed five times by 200 μl extraction buffer. The protein was eluted using 4× SDS loading dye heated for 5 min at 98°C. The eluted protein was loaded onto an SDS-PAGE gel for protein separation.

### Protein expression and purification of tagged protein from *E. coli*

*Escherichia coli* strain BL21 was transformed with MBP fused with OsPSTOL1/OsPSTOL1^2K/R^ in pMAL vector. The positive colony for OsPSTOL1 WT and OsPSTOL1^2K/R^ was inoculated in 10 ml LB cultures containing the appropriate antibiotic (50 μg/ml carbenicillin, Merck, Catalog no. C1613) for 16 h at 37°C. Overnight culture (2.5 ml) was added to a 250-ml LB culture with appropriate antibiotic (50 μg/ml carbenicillin). Two 250-ml cultures were grown at 37°C on a shaker until optical density (O.D.) at 600 nm of the culture was between 0.6 and 0.8. IPTG (Sigma Aldrich, Catalog no. I6758) concentration (1 mM) was used for induction, and cultures were grown at 28°C. Three h after IPTG induction, further samples were taken. All samples were then centrifuged at 10,000 rpm for 5 min. For the total protein extract, the pellet was mixed in 200 μl 1× SDS-PAGE loading buffer. For insoluble and soluble fractions, the pellet was mixed with appropriate volume of bug buster (Merck, Catalog no. 70922-4), according to the weight of the pellet. Cell suspension was incubated on a rotating mixer at slow setting for 20 min. The cell suspension was centrifuged at 13,000 rpm for 30 min at 4°C. The supernatant was transferred into a new microcentrifuge tube. After centrifugation, the pellet was dissolved in 200 μl 1× SDS-PAGE loading dye, and 4× SDS-PAGE loading dye was added to the volume of supernatant collected. A total volume of 20 μl of all samples—uninduced, total protein, soluble, and insoluble fraction, was loaded onto SDS-PAGE to analyse the protein content. The protein was then visualised by immunoblotting. The supernatant was transferred into a new microcentrifuge tube. A volume of 200 μl of the supernatant was separated as an input sample. Equal volume of 2× column buffer with protease inhibitors (40 mM Tris–HCl, pH 7.4, 400 mM NaCl, 2 mM EDTA, and 2 mM PMSF) was added to the supernatant (cell lysate), and the cell lysate was incubated with amylose resin equilibrated with 1× column buffer on an end-to-end rotator at slow speed setting for 2–3 h at 4°C. After the incubation was over, the slurry was passed through a resin retention column, and the flow through was collected in a separate tube. The column was washed twice with 1× column buffer, and the recombinant proteins were eluted from the column with four different maltose (Sigma-Aldrich, Catalog no. 1375025) concentrations—0.5 mM, 1 mM, 3 mM, and 10 Mm—that were prepared in a 1× column buffer. Eight fractions (two elution for each concentration of maltose) were collected. The eluted fractions were loaded on SDS-PAGE and analysed with Coomassie blue staining. Fractions containing recombinant proteins were dialysed overnight in appropriate volumes.

### *In vivo* reconstituted SUMOylation assay in *E. coli*

To perform *in vivo* SUMOylation assay, open reading frames (ORFs) of AtSCE1a and AtSUMO1 were cloned in two different multiple cloning sites (MCS), respectively, in pCDFDuet vector while ORFs of AtSAE1a/b-AtSAE2 were cloned in pACYCDuet vector using the same strategy. *E. coli* BL21 was transformed with pCDFDuet-AtSUMO1 (AA or GG)- AtSCE1a and pACYCDuet-AtSAE1a/b-AtSAE2 plasmids (Okada et al., 2009). After transformation, the cells with these two plasmids were used for the preparation of competent cells. Using this reconstituted SUMOylation system, the difference in SUMOylation status of OsPSTOL1 WT and OsPSTOL1^2K/R^ was investigated.

### *In vitro* and *in planta* phosphorylation assays of OsPSTOL1 protein purified from *E. coli* and rice transgenic lines

To perform the *in vitro* phosphorylation assay, MBP tagged PSTOL1 WT and PSTOL1^2K/R^ and empty vector were transformed into *E. coli* BL21 strain. Protein was purified as described in the method above. The purified proteins were incubated in the kinase reaction buffer for 1 h at 30°C. The reaction was stopped by the addition of 4× loading dye and denatured at 95°C for 5 min. Samples were loaded on both 10% SDS-PAGE and Phos-tag SDS-PAGE.

The YFP-OsPSTOL1 WT and YFP-OsPSTOL1^2K/R^ proteins were immunopurified with anti-GFP antibody beads (Miltenyi Biotech, Catalog no. 130-042-701) as described above from rice transgenic plants. For phosphorylation assays, 50 μl of 1× kinase buffer was added to the column and allowed to run through the column. The supernatant sample (protein bound to GFP beads) was collected in a 1.5-ml microcentrifuge tube. The sample was incubated at 30°C for 1 h. The sample was again run through the column and washed five times with 200 μl extraction buffer. The protein was eluted using 4× SDS loading dye heated for 5 min at 98°C. The eluted protein was loaded onto an SDS-PAGE gel and Phos-tag SDS PAGE for protein separation.

### Confocal microscopy

For *in vivo* plant leaf imaging/roots of rice, leaf sections or root samples were cut (approximately 0.5 cm^2^) and placed on a microscopy slide (Fischer Scientific); a droplet of perfluoroperhydrophenanthrene (PP11) was added before covering the leaf/root section with a 22 × 22 mm cover slip (Menzel-Glaser, Waltham, USA). The slide was placed on the stage of a Zeiss LSM 880 Airyscan confocal microscope (Zeiss, Oberkochen, Germany). A ×63 (objective oil lens) or ×20 was used for viewing, and an argon ion gas laser was used to excite YFP at 514 nm and emission at 524–580 nm.

### Statistical analysis

Data were expressed as mean ± SEM. Statistical differences were tested with ANOVA Tukey test *post-hoc*. Tukey test *post-hoc* was used to compare the mean between three or more groups. p-values <0.05 were considered statistically significant. Statistical analysis was done using software Prism GraphPad.

## Results

### PSTOL1 is conjugated to SUMO1 *in-planta*

**Figure 1:**
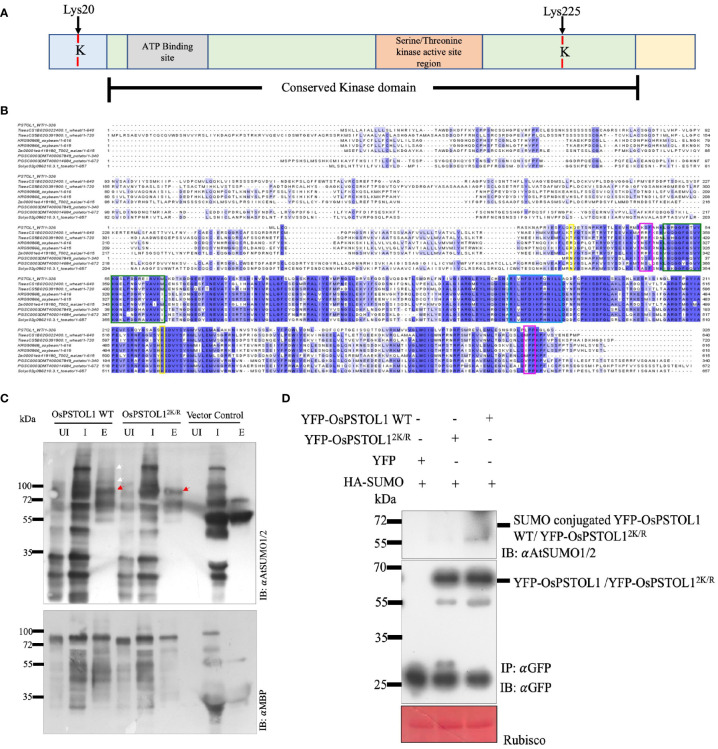
PSTOL1 is a target for SUMO1 modification in plants. **(A)** Predicted SUMO sites were identified in OsPSTOL1 using *in silico* analysis (Verma et al., 2021). The conserved kinase domain is highlighted in green. The serine/threonine kinase domain is depicted in orange. The grey colour shows the ATP-binding domain, and the two SUMO sites are highlighted in red. **(B)** Multiple amino acid sequence alignment of rice OsPSTOL1 with predicted PSTOL1 like protein kinases in wheat, soybean, maize, potato, and tomato. The SUMO sites (Lys 20 and Lys 225) are highlighted in yellow. Start and end of the conserved kinase domain is highlighted in pink. Green highlights ATP binding site and blue highlights serine–threonine kinase domain. **(C)** Immunoblot analysis of *E. coli*-derived SUMOylation assays of OsPSTOL1. N-terminal MBP-tagged OsPSTOL1 WT and OsPSTOL1^2K/R^ proteins were purified using amylose resin from *E. coli* BL21 cells. The uninduced (UI), input (I), and purified protein (E) samples were loaded on SDS-PAGE, and proteins were transferred to a bloating PVDF membrane and probed with α AtSUMO1/2 (IB: αAtSUMO1/2; top panel) and αMBP (IB: αMBP; bottom panel) antibodies. Immunoblot analysis by αAtSUMO1/2 antibody indicated that SUMO conjugates were observed with WT OsPSTOL1, while SUMO conjugates of OsPSTOL1^2K/R^ were not observed, indicating that lysines 20 and 225 are potential SUMO conjugation sites in OsPSTOL1 in *E. coli*. Red arrows indicate molecular weight of MBP-tagged OsPSTOL1, and white arrows indicate bands corresponding to poly-SUMOylation. **(D)** YFP-OsPSTOL1 WT or YFP- OsPSTOL1^2K/R^ was transiently co-expressed with HA-SUMO in leaves of N.benthamiana. Immunoprecipitation (IP: GFP) experiment was carried out with GFP antibody beads from total protein extracted from leaves of N.benthamiana. Immunoblot were probed with GFP (IB: GFP) and AtSUMO1/2 (IB: AtSUMO1/2) antibodies respectivel.

PSTOL1 is a serine–threonine kinase, but how it mediates tolerance to low Pi through its kinase activity is not well understood. Previously, we showed that kinases select their target proteins by the SUMO–SIM interaction module (Verma et al., 2021). Manual interrogation of the OsPSTOL1 protein sequence revealed two lysine residues (Lys20 and Lys225) that could be potential SUMO conjugation sites (**Figures 1A, B**). To understand the role of SUMOylation in mediating OsPSTOL1 mechanism in rice, both potential SUMO sites were mutated to arginine residues (OsPSTOL1^2K/R^), and single SUMO sites mutants (OsPSTOL1^K20R^ and OsPSTOL1^K225R^) constructs were generated using site-directed mutagenesis. SUMOylation status of wild-type OsPSTOL1 (OsPSTOL1 WT) and OsPSTOL1^2K/R^ proteins were first tested in *E. coli* (**Figure 1C**) and subsequently *in planta* (**Supplementary Figure S1**) to ascertain whether the manually interrogated lysine residues were bona fide SUMO conjugation sites. The gene sequences of OsPSTOL1 WT and OsPSTOL1^2K/R^ were cloned into the pMAL vector (c5X) with an N-terminal MBP tag as a translational fusion. Fusion proteins were expressed and purified from *E. coli* cultures. *In vitro* reconstituted SUMOylation assays (Okada et al., 2009) demonstrated conjugation of *Arabidopsis* AtSUMO1 to OsPSTOL1 WT, while conjugation was not seen in OsPSTOL1^2K/R^ (**Figure 1C**). Furthermore, to ascertain OsPSTOL1 SUMOylation status *in planta*, *Agrobacterium*-mediated transient assay in *N. benthamiana* performed using 35S CAMV promoter driven YFP-tagged OsPSTOL1 WT (35S::YFP-OsPSTOL1) and double SUMO site lysine mutated OsPSTOL1^2K/R^ (35::YFP-OsPSTOL1^2K/R^), single SUMO site mutant OsPSTOL1^K20R^ (35::YFP- OsPSTOL1^K20R^), and OsPSTOL1^K225R^ (35::YFP- OsPSTOL1^K225R^) DNA constructs were co-expressed, respectively, with an HA-tagged AtSUMO1 (35S::HA-SUMO). Recombinant YFP-OsPSTOL1 WT, YFP-OsPSTOL1^K20R^, YFP-OsPSTOL1^K225R^, and YFP-OsPSTOL1^2K/R^ expression was confirmed by Western blotting with αGFP antibodies (**Supplementary Figure S1**). Our data indicate that there is almost undetectable SUMOylation of YFP-PSTOL1^2K/R^ when compared to YFP-OsPSTOL1 WT or the single SUMO sites mutants of PSTOL1 *in planta*, indicating that both lysine residues are important for SUMO conjugation (**Supplementary Figure S1**). However, it appears that SUMO conjugation to PSTOL1 is more severely hampered when lysine 225 of PSTOL1 is mutated to arginine than when lysine 20 is mutated to arginine, therefore indicating that K225 is a major site for SUMO conjugation in the *N. benthamiana*. Finally, our data also indicated that in the *E. coli* system, OsPSTOL1 SUMOylation resolves into multiple bands but exhibits a single band at approximately 65 kDa *in planta* (**Figure 1D**).

### SUMO conjugation to OsPSTOL1 plays a vital role in regulating autophosphorylation activity of OsPSTOL1

**Figure 2:**
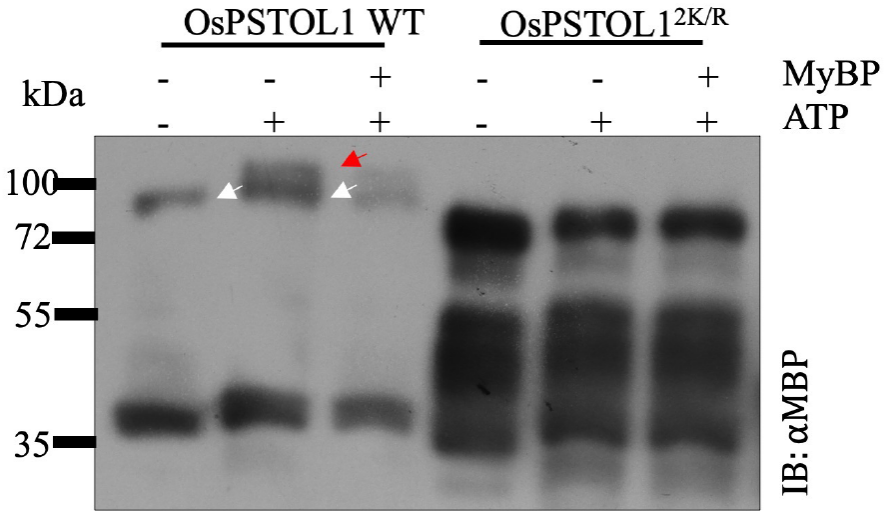
SUMOylation of OsPSTOL1 plays a vital role in regulating its kinase activity. Immunoblot analysis of the phosphorylation activity of OsPSTOL1 WT and non-SUMOylatbale OsPSTOL1^2K/R^. Purified N-terminal MBP-tagged bacterial proteins OsPSTOL1 WT and OsPSTOL1^2K/R^ were incubated with and without myelin basic protein (MyBP) in kinase reaction mixture (including ATP) to determine cross- phosphorylation and autophosphorylation activity, respectively. The Western blot represents samples run on Mn^+2^ Phos-tag gels with 5μM of Phos-tag acrylamide and 10μM MnCl2. The red arrow indicates the phosphorylated form of the protein above 80kDa, and the white arrow indicates the dephosphorylated form of protein at expected size of 80kDa.

After confirming that OsPSTOL1 is SUMOylated, the next step was to ascertain how SUMOylation affects PSTOL1 kinase activity. An *in vitro* phosphorylation assay was performed to investigate the OsPSTOL1/OsPSTOL1^2K/R^ autophosphorylation activity. The Phos-tag gel analysis of MBP-tagged OsPSTOL1 WT and OsPSTOL1^2K/R^ protein purified from *E. coli* incubated without kinase buffer showed as a single band at 80 kDa (**Figure 2**). However, when OsPSTOL1 WT protein was incubated with ATP, the WT protein was separated into two different forms because of altered mobility of phosphorylated (**Figure 2**, indicated by red arrow) and non-phosphorylated (**Figure 2**, indicated by white arrow) forms, indicating autophosphorylation activity of OsPSTOL1 WT. The incubation of OsPSTOL1 WT protein with both ATP and myelin basic protein (MyBP) showed less intensity of both phosphorylated and dephosphorylated form of PSTOL1 WT, suggesting an increased trans-phosphorylation of the substrate MyBP by PSTOL1 WT kinase with a concomitant decrease in autophosphorylation. However, we could not detect any autophosphorylation or trans-phosphorylation activity of OsPSTOL1^2K/R^ (**Figure 2**). Additionally, to determine whether there is complete loss of autophosphorylation activity in OsPSTOL1^2K/R^, we loaded more OsPSTOL1^2K/R^ protein compared to OsPSTOL1 WT to confirm any remaining autophosphorylation activity of OsPSTOL1^2K/R^. As shown in **Figure 2**, we did not observe any residual autophosphorylation activity in samples containing non-SUMOylatable version of OsPSTOL1; however, overloading the lanes in all three lanes of OsPSTOL1^2K/R^ has caused greater migration of MBP-OsPSTOL1^2K/R^ in SDS-PAGE, therefore appearing to be smaller than its expected size. Nevertheless, our data indicate that SUMOylation has a fundamental role in regulating the autophosphorylation activity of OsPSTOL1.

### SUMOylation of OsPSTOL1 regulates total root length and total root surface area of rice roots under low Pi

**Figure 3:**
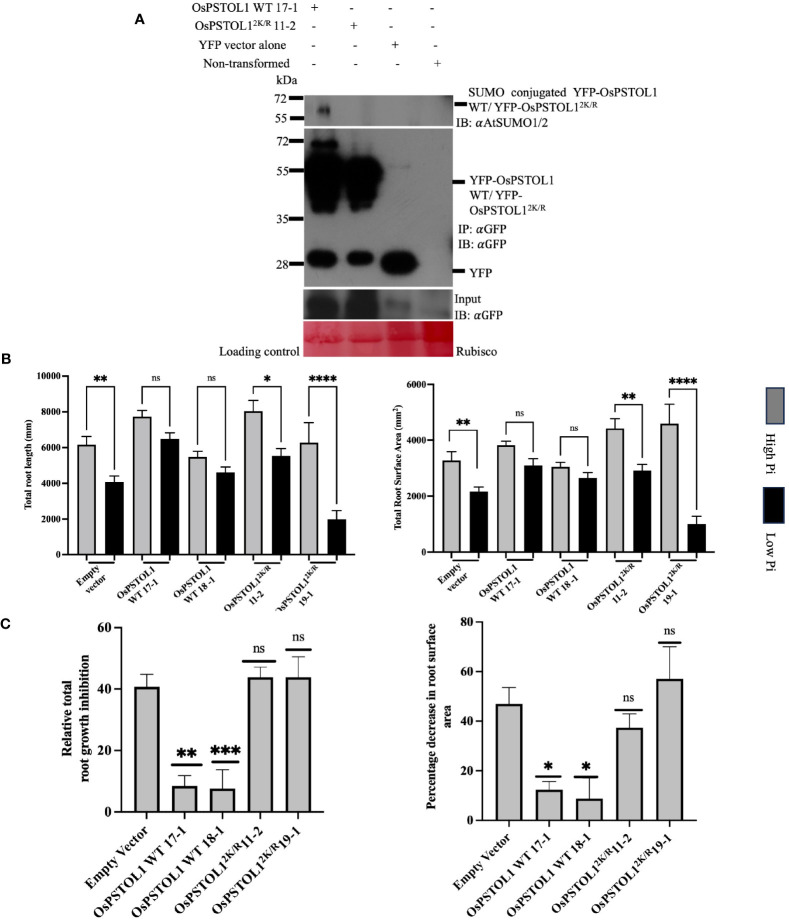
SUMOylation of OsPSTOL1 is essential for maintaining root system architecture (RSA) of rice plants under low Pi. **(A)** Immunoblot analysis of immunoprecipitation experiment of YFP-OsPSTOL1 WT, YFP-OsPSTOL1^2K/R^, YFP vector alone, and empty vector was carried out with αGFP antibody beads. Total protein was extracted from 10-old-day rice seedlings. Western blot was probed with αGFP (IB: αGFP) and αAtSUMO1/2 (IB: αAtSUMO1/2) antibodies, respectively. YFP vector and Nipponbare seedlings (untransformed) were used as negative control. Ponceau stained for RuBisCO is shown as a loading control for the experiment. **(B)** Analysis of total root length and total root surface area of roots of 28-days-old overexpressing UBI::YFP-OsPSTOL1 WT and UBI::YFP-OsPSTOL1^2K/R^ transgenic plants with corresponding non-transformed (Nipponbare) under high Pi (100μM) and low Pi (3μM). Error bars represent ± SEM (n=16) of three independent biological replicates. Statistical differences were calculated using ordinary one-way ANOVA Tukey’s multiple comparison test. ****p < 0.0001, **p = 0.0012, *p = 0.05 significant; ns, not significant, significance represent between treated and non-treated. **(C)** Analysis of relative root growth and percentage decrease in root surface area of overexpressing OsPSTOL1 WT/OsPSTOL1^2K/R^ rice transgenic lines under low Pi. Error bars represent + SEM (n=16) of three biological replicates. Statistical differences were calculated using ordinary one-way ANOVA Tukey’s multiple comparison test. *p < 0.05, **p = 0.0011, ***p = 0.0002 and ns, not significant, significance represent between empty vector and transgenic lines.

Previous data suggested that overexpression of OsPSTOL1 kinase in IR64 and Nipponbare plants results in improved grain yield by 60% when rice plants are grown in Pi-limited soil (Gamuyao et al., 2012). This increase in overall grain yield is hypothesised to be due to enhanced root development in low-phosphorus soil, thereby improving the plant’s ability to “mine” phosphorus from soil (Gamuyao et al., 2012). Nevertheless, the regulatory mechanism underpinning this effect is still elusive. To ascertain if SUMO plays a role in regulating OsPSTOL1-dependent enhanced root development and thereby promote phosphorus starvation tolerance in rice, we generated OsPSTOL1 WT and OsPSTOL1^2K/R^ C-terminal YFP-tagged overexpressing rice transgenic lines under the control of a maize ubiquitin promoter in Nipponbare rice variety. We ascertained the gene copy number of transgenic lines by qPCR using the relative quantification method that determines the copy number of a transgene by comparing the transcript level of a transgene (in this case hygromycin) to that of an endogenous reference gene (in this case sucrose–phosphate synthase, SPS) with a determined copy number (Shepherd et al., 2009; Xiujie et al., 2019) (**Supplementary Figure S2A**). The mRNA levels of single-copy transgenic rice lines were also determined (**Supplementary Figure S2B**, **Supplementary Table S1**). Furthermore, total protein was extracted in the selected single-copy transgenic rice lines to ascertain expression levels of OsPSTOL1 protein levels (**Supplementary Figure S3**). Western blot analysis showed similar protein levels in OsPSTOL1 WT 17-1, OsPSTOL1 WT 18-1, OsPSTOLL1^2K/R^11-2, and OsPSTOLL1^2K/R^19-1. These independent homozygous lines from each genotype were selected for further analysis. Next, we investigated the SUMOylation status of YFP-OsPSTOL1 WT and YFP-OsPSTOL1^2K/R^ in rice transgenics with similar OsPSTOL1 protein levels to assess whether any Pi tolerance phenotype observed is due to difference in SUMOylation status of YFP-OsPSTOL1 WT and YFP-OsPSTOL1^2K/R^. **Figure 3A** shows the result of the immunoblot analysis with αGFP antibody (IB:αGFP) where OsPSTOL1 WT and OsPSTOL1^2K/R^ tagged with YFP can be detected. Interestingly, we observed SUMO conjugated to YFP-OsPSTOL1 WT, while this conjugation was significantly reduced in YFP-OsPSTOL1^2K/R^ when the immunoprecipitated samples were analysed with αAtSUMO1/2 antibodies (**Figures 3A**, upper panel), which further substantiate our previous *in planta* analysis from **Supplementary Figure S1**.

Once the SUMOylation status of OsPSTOL1 in rice transgenic lines was confirmed, our next aim was to investigate the role of SUMOylation of OsPSTOL1 in regulating root growth and development. The elongation of primary root length and reduction in mean diameter, total root length, and total root surface area of Nipponbare roots are some typical phenotypic traits observed in response to Pi starvation (Wissuwa and Ae, 2001; Zhou et al., 2008; Vejchasarn et al., 2016; Deng et al., 2020). To study the phosphate-dependent responses in roots of rice, plants containing empty vector and transformed plants expressing YFP-OsPSTOL1 WT and YFP-PSTOL1^2K/R^ were grown under high (100 μM) and low (3μM) Pi conditions in the greenhouse, and the plants were phenotypically assessed after 28 days of growth. To evaluate the effect of phosphate availability on root development, we assessed total root length and total root surface area. Our data revealed that under high Pi conditions, there was no statistically significant difference in total root length and total root surface area between empty vector and OsPSTOL1 WT/OsPSTOL1^2K/R^ transgenic lines. However, in low Pi conditions, the total root length growth and overall root surface area were significant less inhibited (13%–19%) in OsPSTOL1 WT rice, while empty vector and OsPSTOL1^2K/R^ showed significant growth inhibition (~33%–77%) when compared to the plants grown in Pi-replete conditions (**Figures 3B, C**). Therefore, our data revealed that SUMOylation of OsPSTOL1 is required to enhance total root growth in rice as an adaptive response to improve tolerance under Pi-deficient conditions.

**Figure 4:**
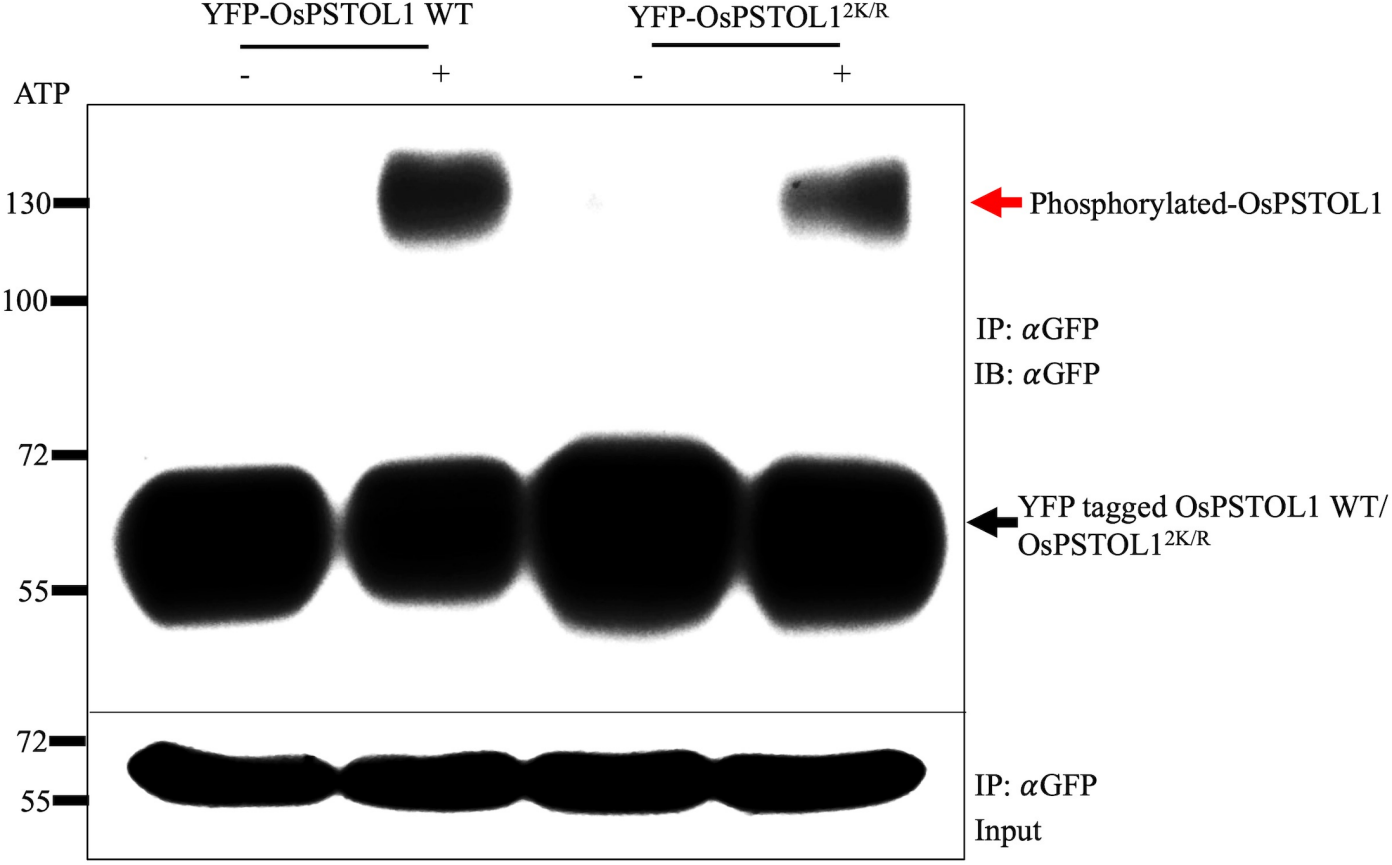
SUMOylation is essential for autophosphorylation activity of OsPSTOL1 in rice transgenic seedlings. YFP-tagged OsPSTOL1 WT and non-SUMMO mutant OsPSTOL1^2K/R^ protein were isolated from rice transgenic lines and incubated with kinase reaction mixture with and without ATP to determine autophosphorylation activity. The Western blot represents samples run on Mn^+2^ Phos-tag gels with 5μM of Phos-tag acrylamide and 10μM MnCl2 (upper panel). The lower panel indicates input gel for protein loading. The red arrow indicates the phosphorylated form of the protein at 130kDa, and the black arrow indicates the dephosphorylated form of protein at expected size of 65kDa.

Furthermore, to confirm whether the reduced autophosphorylation activity of non-SUMO OsPSTOL1 observed *in vitro* also occurs *in planta*, we performed autophosphorylation assays with OsPSTOL1 immunopurified from rice transgenic plants. YFP-tagged OsPSTOL1 WT and non-SUMO mutants OsPSTOL1^2K/R^ protein were isolated from rice transgenic lines and incubated with kinase reaction mixture with and without ATP to determine autophosphorylation activity. Our data indicated that non-SUMO mutants OsPSTOL1^2K/R^ protein has reduced autophosphorylation activity (**Figure 4**, upper panel) when compared to WT protein, therefore demonstrating that SUMOylation of OsPSTOL1 is important for its kinase activity. Hence, we were able to form a biochemical link between SUMOylation, kinase activity of OsPSTOL1, and root growth inhibition in rice under low Pi conditions.

### Overexpression of OsPSTOL1 in *Arabidopsis* showed enhanced root growth under low Pi conditions

**Figure 5:**
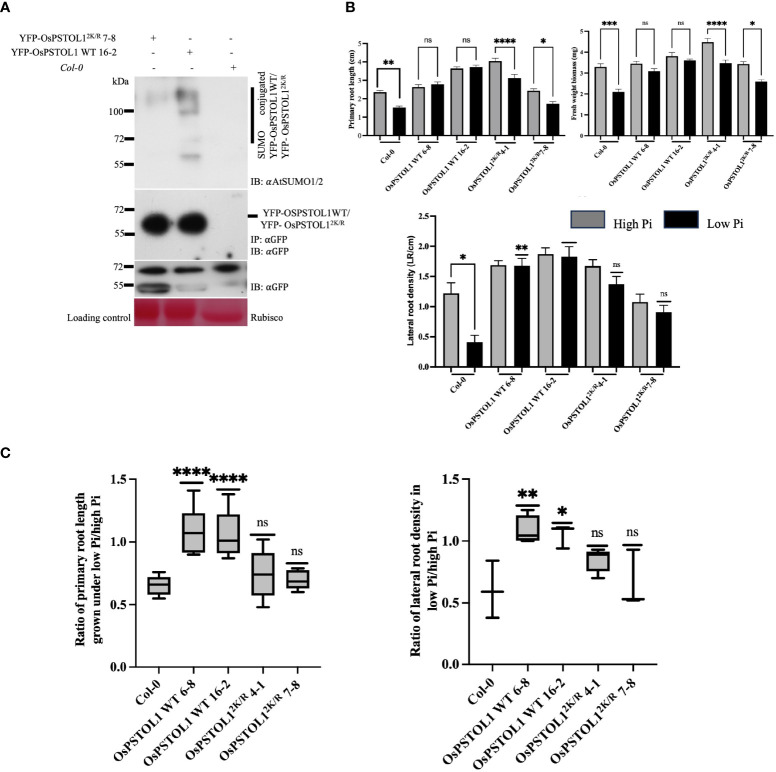
Overexpression of OsPSTOL1 in heterologous system, *Arabidopsis* increases root growth under Pi starvation conditions. **(A)** Immunoblot analysis of immunoprecipitation experiments of YFP-OsPSTOL1 WT, YFP-OsPSTOL12^K/R^, and *Col-0* (negative control) was carried out with αGFP antibody beads. Total protein was extracted from 14-old-day rice seedlings. αAtSUMO1/2 (IB: αAntiSUMO 1/2) and αGFP antibodies (IB: αGFP) were used, respectively. *Col-0* was used as negative control. Ponceau stained for RuBisCO is shown as a loading control for the experiment. **(B)** Quantification of root length, lateral root density, and fresh weight biomass of *Col-0*, OsPSTOL1 WT 6-8, OsPSTOL1 16-2, OsPSTOL12^K/R^ 4-1, and OsPSTOL12^K/R^ 7-8 on high (1.25mM) and low phosphate (3μM). Four-day-old seedlings of different genotypes were transferred to MS with high (1.25mM) and low phosphate (3μM) and grown for additional 11 days until primary root length, lateral root density, and fresh weight biomass were measured. Data for primary root length and fresh weight biomass were averaged as SD ± SEM (n = 40) of three independent biological replicates. Statistical differences was calculated using ordinary one-way ANOVA Tukey’s multiple comparison test. ****p < 0.0001, ***p = 0.0002, **p = 0.0092, *p = 0.02 significant; ns, not significant, significance represent between treated and non-treated seedlings. Data for lateral root density were averaged as SD ± SEM (n = 40) of three independent biological replicates. Statistical differences was calculated using ordinary one-way ANOVA Tukey’s multiple comparison test and Welch test (indicating the comparison between *Col-0* seedlings grown on high and low Pi condition). **p = 0.002, ***p = 0.0002; ns, not significant, significance represents comparison between with wild-type subjected to low Pi treatment (*Col-0*). **(C)** Analysis of ratio of primary root growth and lateral root density of overexpressing OsPSTOL1 WT/OsPSTOL1^2K/R^ *Arabidopsis* transgenic seedlings under low- and high-Pi conditions. Error bars represent ± SEM (n=40) of three independent biological replicates. Statistical differences were calculated using ordinary one-way ANOVA Tukey’s multiple comparison test. ***p = 0.0009–0.0010, **p = 0.0093, *p = 0.024 significant; ns, not significant and significance represent ratio between empty vector and transgenic lines.

Given the importance of OsPSTOL1 in rice, we wanted to ascertain its role in the model plant *Arabidopsis* and identify any conserved molecular mechanisms of low Pi tolerance mediated through OsPSTOL1 even though this gene is not present in the genome of *Arabidopsis thaliana*. Understanding fundamental molecular mechanisms underpinning Pi starvation responses using fast-cycling *Arabidopsis thaliana* will allow rapid translation of new findings in other non-cereal crop plants. In *Arabidopsis*, the limitation of Pi causes root architecture modifications such as inhibition of primary root length, increase in lateral root density/length, root hair density/length, and increase root/shoot ratio (Miura et al., 2005; Huang and Zhang, 2020). Transgenic lines overexpressing OsPSTOL1 WT and OsPSTOL1^2K/R^ with N-terminal YFP fusions were generated, and these lines were analysed for their root development responses under high and low Pi. Real-time PCR analysis of *YFP-OsPSTOL1 WT* and *YFP-OsPSTOL1^2K/R^* indicated consistent gene expression in at least two independent transgenic lines from each genotype—OsPSTOL1 WT 6-8, OsPSTOL1 WT 16-2, OsPSTOL1^2K/R^ 4-1, and OsPSTOL1^2K/R^ 7-8 (**Supplementary Figure S4**, **Supplementary Table S1**), and furthermore, equivalent protein expression in these independent lines of YFP-OsPSTOL1 WT and YFP-OsPSTOL1^2K/R^ was also confirmed using Western blot analysis (**Supplementary Figure S5**). Next, we again analysed the SUMOylation status of YFP-OsPSTOL1 WT and YFP-OsPSTOL1^2K/R^ in *Arabidopsis* transgenics lines. Our data confirmed that SUMOylation of OsPSTOL1 occurred in *Arabidopsis*, and YFP-OsPSTOL1^2K/R^ showed significantly reduced SUMOylation (**Figure 5A**). This result further validates our previous results of SUMOylation status of OsPSTOL1 WT/OsPSTOL1^2K/R^ from *in vivo* reconstituted SUMOylation assay, transient assay in *N. benthamiana*, and rice transgenic lines.

To analyse root development of *Arabidopsis* transgenic lines expressing OsPSTOL1 WT/OsPSTOL1^2K/R^, we grew these lines in high (1.25 mM) and low (3μM) Pi, and plant growth phenotypes were assessed after 11 days. We observed that YFP-OsPSTOL1 WT transgenic lines under the 35S constitutive promoter displayed improved performance than untransformed *Col-0* plants in three growth parameters—primary root length, lateral root density, and fresh weight—of seedlings in response to high and low Pi treatments. Interestingly, YFP-OsPSTOL1 WT transgenic lines exhibited increased root length in Pi starvation. We observed that YFP-OsPSTOL1 WT transgenic lines showed a 2.1%–5.3% increase in primary root length in low Pi when compared to high Pi. Further analysis also indicated that primary root length was significantly reduced in YFP-OsPSTOL1^2K/R^ transgenic lines (22.7%–29%) similar to *Col-0* (21.2%) under Pi-starved conditions (**Figures 5B, C**). Another important parameter that we analysed was lateral root density. As indicated in **Figure 5B**, OsPSTOL1 WT transgenic lines exhibited significantly increased lateral root density in low Pi conditions than *Col-0* or transgenics expressing OsPSTOL1^2K/R^, which is reflected furthermore when we assessed the ratio of lateral root density between *Col-0*, OsPSTOL1 WT, and OsPSTOL1^2K/R^ lines grown in low Pi conditions versus high Pi conditions (**Figure 5C**).

Pi limitation profoundly affects overall growth and development of plants (Shin et al., 2004), so to determine whether low availability of Pi affects overall plant growth in OsPSTOL1 transgenic lines, we measured fresh weight of *Arabidopsis* seedlings grown on high and low Pi medium. Our data indicate that the fresh weight gain of OsPSTOL1 WT transgenic lines is less inhibited compared to OsPSTOL1^2K/R^ (**Figure 5B**). Taken together, the results demonstrate an important positive regulatory role of SUMOylation of OsPSTOL1 in mediating plant growth during Pi deficiency.

### OsPSTOL1 WT/OsPSTOL1^2K/R^ localises in the nuclei and cytoplasm in leaves of *N. benthamiana*, roots of rice, and *Arabidopsis* transgenic lines

**Figure 6:**
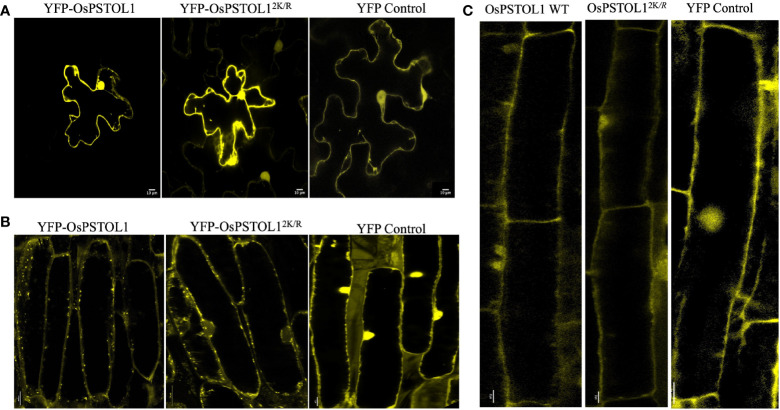
OsPSTOL1 localises in nucleus and cytoplasm of the cell in tobacco leaves and roots of rice and *Arabidopsis* plants. YFP-tagged OsPSTOL1 WT/OsPSTOL1^2K/R^ localises in nucleus and cell cytoplasm when expressed in **(A)** *N. benthamiana* leaves, **(B)** roots of rice transgenic lines overexpressing OsPSTOL1 WT/OsPSTOL1^2K/R^, and **(C)** Roots of *Arabidopsis* OsPSTOL1 WT transgenic lines. The localisation was analysed by Zeiss LSM 880 confocal laser scanning microscope using a 60 × 1.4NA lens (for observing OsPSTOL1 WT/OsPSTOL1^2K/R^ in *N. benthamiana* leaves and roots of rice transgenic lines) and ×20 lens for observing OsPSTOL1 expression roots of *Arabidopsis* transgenic lines using laser excitation for YFP at 514 nm and emission filters of 524–580 nm. Scale bar: 10*μ*m.

Due to the differences in root development in rice and *Arabidopsis*, we wanted to ascertain whether OsPSTOL1 protein was targeted to the same subcellular location in roots of rice and *Arabidopsis* transgenic lines and additionally in leaves of *N. benthamiana*. Therefore, we performed confocal microscopy analysis expressing YFP-OsPSTOL1 WT and YFP-OsPSTOL1^2K/R^ in three different systems. Our data indicate that OsPSTOL1 localises to the nucleus and cytoplasm in leaves of tobacco plants and roots of *Arabidopsis* and rice transgenic plants (**Figures 6A–C**).

## Discussion

Proteins are subjected to various post-translational modification (PTMs), which significantly increase the functionality and diversity of the proteome, improving the ability of plants to respond to abiotic or biotic stresses. As a result, plants can fine-tune responses, and studying these responses is an important aspect of crop improvement efforts (Zhang and Zeng, 2020). Our results demonstrated that mutating lysines 20 and 225 in OsPSTOL1 can significantly reduce SUMO conjugation *in vivo* and *in planta*, indicating that they are bona fide SUMO sites (*N. benthamiana*, *Arabidopsis*, and rice). In recent years, it is well established that cross-talk between different post-translational modifications will regulate protein activity and its fate (Verma et al., 2021). SUMOylation of WRKY33 is necessary to interact with mitogen-activated protein kinases (MAPKs), which results in phosphorylation of WRKY33 and activation (Verma et al., 2021). This emerging evidence add weights to the argument that cross-talk between different PTMs allow additional layers of regulation and thus fine-tuning plant responses to environmental cues (Zhang and Zeng, 2020). We investigated crosstalk between SUMOylation and phosphorylation, and we demonstrated that SUMO sites (Lys20 and Lys225) in OsPSTOL1 play a vital role in regulating autophosphorylation and transphosphorylation *in vitro*. The data suggest that SUMOylation is the PTM-regulating basal-level PSTOL1 kinase activity, and SUMOylation may play a regulatory role in substrate selectivity of PSTOL1 kinase during normal growth conditions and stress conditions, therefore linking phosphorylation activity of PSTOL1 with SUMOylation.

Low Pi stress affects overall growth of plants such as reduced total surface area, total root length, and plant height. Plants have evolved several adaptive responses to improve and adapt to low Pi conditions. Alteration of root architecture is one of the most important adaptions to low Pi by plants because root architecture is extremely plastic in response to differences in phosphate conditions (Huang and Zhang, 2020). However, dicot (*Arabidopsis thaliana*) and monocot (rice) species have different regulatory mechanisms to adapt to low-Pi stress. Rice being a monocot plant has developed fibrous root systems showing elongation of primary root length. Pi deficiency induces shallower root system by increasing root hair formation and length and enhances lateral root density or crown root growth, but attenuates primary root growth in dicots such as *Arabidopsis*. Increased root hair and lateral root number leads to greater root surface area for absorption of Pi during Pi-starved conditions. Increase in total root growth and shallower root system facilitate the plants’ ability to acquire low mobility Pi from the topsoil, a strategy referred to as “topsoil foraging” (Miura et al., 2011; Sato and Miura, 2011; Huang and Zhang, 2020; Pandey et al., 2021; Prathap et al., 2022). In this study, we showed that SUMOylation of OsPSTOL1 is essential for regulating root system architecture in low Pi availability, and SUMOylation has an important role in regulating root adaptative responses in Pi deficiency. Reports from Wissuwa and Ae (2001) and Gamuyao et al. (2012) had showed that Pi limitation significantly reduces total surface area and total root length of Nipponbare plants. Under Pi-deficient conditions, empty vector and OsPSTOL1^2K/R^ transgenic lines showed reduction in these two parameters. In contrast, OsPSTOL1 WT transgenic lines was unaffected by Pi supply. We postulate that this is due to the increase in the number of lateral roots that eventually increase total surface area and total root length. However, both genotypes were indistinguishable and have greater total surface area and total root length in comparison to empty vector in Pi-replete conditions.

The functional characterisation of OsPSTOL1 WT and OsPSTOL1^2K/R^ was further investigated by expressing the proteins in a heterologous system, *Arabidopsis thaliana*. The Pi-concentration-dependent analysis of 35S::YFP OsPSTOL1 WT and 35S::YFP-OsPSTOL1^2K/R^ *Arabidopsis* transgenic lines indicates that overexpression of OsPSTOL1 WT in *Arabidopsis* showed enhanced root growth, more lateral root density, and increased fresh weight biomass when compared to vector-control plants, which provides strong evidence of OsPSTOL1 WT acting to boost root development irrespective of Pi supply which has been substantiated by our data analysing OsPSTOL1 rice transgenic plants in high and low Pi. On the other hand, OsPSTOL1^2K/R^ transgenic lines exhibited reduced primary root length, decreased lateral root density, and eventual decrease in fresh weight biomass in Pi-starved conditions. This phenotype observed in YFP-OsPSTOL1^2K/R^ lines could probably be because of two reasons: either the formation of new cells is reduced in root apical meristem or a significant reduction in cell expansion in the elongation zone or a combination of both. Another possible hypothesis is that PSTOL1 might phosphorylate ARF7 and ARF19, which are important genes for lateral root initiation under Pi starvation in *Arabidopsis* (Huang et al., 2018), and these ARFs are subjected to various post-translational modifications. Cho et al. (2014) showed that phosphorylation of ARF7 and ARF19 mediated by BIN2 (BRASSINOSTEROID INSENSITIVE 2) is an essential step to initiate the lateral root organogenesis by increasing the transcription activity of *LATERAL ORGAN BOUNDARIES-DOMAIN16 (LBD16)* and *LATERAL ORGAN BOUNDARIES-DOMAIN29 (LBD29)*. OsPSTOL1 could potentially phosphorylate *ARF7* and *ARF19*, thereby activating these ARFs in responses to Pi starvation. Our *in vitro* kinase assay reveals the loss of autophosphorylation activity of the non-SUMOylatable version of OsPSTOL1. This prompted us to hypothesise that non-SUMOylatable version of OsPSTOL1 probably cannot phosphorylate these ARFs, which ultimately results in altered root architecture under low Pi.

## Conclusions

Taken together, we have found that overexpressing OsPSTOL1 in Nipponbare have maintained the total root surface area and total root length under low Pi to conserve the metabolic cost of topsoil foraging, while overexpression of non-SUMOylatable version of OsPSTOL1 have reduced total root surface area and total root length under low Pi. Furthermore, functional characterisation of OsPSTOL1 in *Arabidopsis thaliana* also showed that the expression of OsPSTOL1 does promote root development by promoting primary root length, enhancing lateral root density and fresh weight biomass, while *Arabidopsis* transgenic plants overexpressing OsPSTOL1^2K/R^ did not demonstrate enhanced root growth. The results in rice and *Arabidopsis* show that SUMO underpins a conserved core stress response pathway in model plants and crops affecting root development. Although phenotypic analysis does indicate a conserved regulatory network, nevertheless, components of this network are yet to be identified in crops and model plants. In addition, we also demonstrated the localisation of OsPSTOL1 in roots of rice and *Arabidopsis* and leaves of *N. benthamiana*. Lastly, as OsPSTOL1 is a kinase, we demonstrated that SUMOylation is the PTM governing kinase activity of OsPSTOL1.

## Data availability statement

The original contributions presented in the study are included in the article/**Supplementary material**, further inquiries can be directed to the corresponding author.

## Author contributions

AS: Conceptualization, Funding acquisition, Investigation, Methodology, Project administration, Resources, Supervision, Writing – original draft, Writing – review & editing. VM: Conceptualization, Formal analysis, Methodology, Writing – original draft, Writing – review & editing. DR: Formal analysis, Investigation, Methodology, Writing – review & editing. CZ: Formal analysis, Investigation, Methodology, Writing – review & editing. KS-O: Formal analysis, Methodology, Writing – review & editing. AJ: Writing – review & editing. PKB: Writing – review & editing. SK: Writing – review & editing. SH: Writing – review & editing.

## References

[B1] Carlos Barragán-Rosillo A. Barragán-Rosillo A.C. Peralta-Alvarez C.A. Ojeda-Rivera J. O. Arzate-Mejía R. G. Recillas-Targa F. (2021). Genome accessibility dynamics in response to phosphate limitation is controlled by the PHR1 family of transcription factors in Arabidopsis. Proc. Natl. Acad. Sci. 118 (33), e2107558118. doi: 10.1073/pnas.2107558118 34385324 PMC8379931

[B2] Cho H. Ryu H. Rho S. Hill K. Smith S. Audenaert D.. (2014). A secreted peptide acts on BIN2-mediated phosphorylation of ARFs to potentiate auxin response during lateral root development. Nat. Cell Biol. 16 (1), 66–76. doi: 10.1038/ncb2893 24362628

[B3] Clark L. Sue-Ob K. Mukkawar V. Jones A. R. Sadanandom A. (2022). “Understanding SUMO-mediated adaptive responses in plants to improve crop productivity,” in Essays in Biochemistry (United Kingdom: Portland Press Ltd), 155–168. doi: 10.1042/EBC20210068 PMC9400072 35920279

[B4] Clough S. J. Bent A. F. (1998). Floral dip: A simplified method for Agrobacterium-mediated transformation of Arabidopsis thaliana. Plant J. 16, 735–743. doi: 10.1046/j.1365-313x.1998.00343.x 10069079

[B5] Crombez H. Motte H. Beeckman T. (2019). “Tackling plant phosphate starvation by the roots,” in Developmental Cell (United Kingdom: Cell Press), 599–615. doi: 10.1016/j.devcel.2019.01.002 30861374

[B6] Deng S. Lu L. Li J. Du Z. Liu T. Li W.. (2020). Purple acid phosphatase 10c encodes a major acid phosphatase that regulates plant growth under phosphate-deficient conditions in rice. J. Exp. Bot. 71 (14), 4321–4332. doi: 10.1093/jxb/eraa179 32270183 PMC7475256

[B7] Gamuyao R. Chin J. H. Pariasca-Tanaka J. Pesaresi P. Catausan S. Dalid C.. (2012). The protein kinase Pstol1 from traditional rice confers tolerance of phosphorus deficiency. Nature 488 (7412), 535–539. doi: 10.1038/nature11346 22914168

[B8] Heuer S. Lu X. Chin J. H. Tanaka J. P. Kanamori H. Matsumoto T.. (2009). Comparative sequence analyses of the major quantitative trait locus phosphorus uptake 1 (Pup1) reveal a complex genetic structure. Plant Biotechnol. J. 7 (5), 456–471. doi: 10.1111/j.1467-7652.2009.00415.x 19422603

[B9] Hiei Y. Komari T. (2008). Agrobacterium-mediated transformation of rice using immature embryos or calli induced from mature seed. Nat. Protoc. 3, 824–834. doi: 10.1038/nprot.2008.46 18451790

[B10] Huang G. Zhang D. (2020). “The plasticity of root systems in response to external phosphate,” in International Journal of Molecular Sciences (United Kingdom: MDPI AG), 1–12. doi: 10.3390/ijms21175955 PMC7503333 32824996

[B11] Huang K. L. Ma G. J. Zhang M. L. Xiong H. Wu H. Zhao C. Z.. (2018). The ARF7 and ARF19 transcription factors positively regulate PHOSPHATE STARVATION RESPONSE1 in Arabidopsis roots. Plant Physiol. 178 (1), 413–427. doi: 10.1104/pp.17.01713 30026290 PMC6130041

[B12] Lott J. N. A. Kolasa J. Batten G. D. Campbell L. C. (2011). The critical role of phosphorus in world production of cereal grains and legume seeds. Food Secur. 3, 451–462. doi: 10.1007/s12571-011-0144-1

[B13] Miura K. Lee J. Gong Q. Ma S. Jin J. B. Yoo C. Y. (2011). SIZ1 Regulation of phosphate starvation-induced root architecture remodeling involves the control of auxin accumulation. Plant Physiol. 155 (2), 1000–1012. doi: 10.1104/pp.110.165191 21156857 PMC3032448

[B14] Miura K. Rus A. Sharkhuu A. Yokoi S. Karthikeyan A. S. Raghothama K. G.. (2005). The Arabidopsis SUMO E3 ligase SIZ1 controls phosphate deficiency responses. Proc. Natl. Acad. Sci. 102 (21), 7760–7765. doi: 10.1073/pnas.0500778102 15894620 PMC1140425

[B15] Neelam K. Thakur S. Neha Yadav I. S. Kumar K. Dhaliwal S. S. Singh K.. (2017). Novel alleles of phosphorus-starvation tolerance 1 gene (PSTOL1) from oryza rufipogon confers high phosphorus uptake efficiency. Front. Plant Sci. 8, 509. doi: 10.3389/fpls.2017.00509 28443109 PMC5387083

[B16] Okada S. Nagabuchi M. Takamura Y. Nakagawa T. Shinmyozu K. Nakayama J. I.. (2009). Reconstitution of Arabidopsis thaliana SUMO pathways in E. coli: Functional evaluation of SUMO machinery proteins and mapping of SUMOylation sites by mass spectrometry. Plant Cell Physiol. 50 (6), 1049–1061. doi: 10.1093/pcp/pcp056 19376783

[B17] Pandey B. K. Verma L. Prusty A. Singh A. P. Bennett M. J. Tyagi A. K.. (2021). OsJAZ11 regulates phosphate starvation responses in rice. Planta 254 (1), 8. doi: 10.1007/s00425-021-03657-6 34143292 PMC8213676

[B18] Pei W. Jain A. Zhao G. Feng B. Xu D. Wang X. (2020). Knockdown of OsSAE1a affects the growth and development and phosphate homeostasis in rice. J. Plant Physiol. 255, 153275. doi: 10.1016/j.jplph.2020.153275 33161338

[B19] Prathap V. Kumar A. Maheshwari C. Tyagi A. (2022). “Phosphorus homeostasis: acquisition, sensing, and long-distance signaling in plants,” in Molecular Biology Reports (United Kingdom: Springer Science and Business Media B.V.), 8071–8086. doi: 10.1007/s11033-022-07354-9 35318578

[B20] Qazi H. A. Jan N. Ramazan S. John R. (2019). “Protein modification in plants in response to abiotic stress,” in Protein Modificomics: From Modifications to Clinical Perspectives (United Kingdom: Elsevier), 171–201. doi: 10.1016/B978-0-12-811913-6.00008-4

[B21] Sato A. Miura K. (2011). “Root architecture remodeling induced by phosphate starvation,” in Plant Signaling and Behavior (United Kingdom: Landes Bioscience), 1122–1126. doi: 10.4161/psb.6.8.15752 PMC3260708 21778826

[B22] Shepherd C. T. Lauter A. N. M. Scott M. P. (2009). Determination of transgene copy number by real-time quantitative PCR. Methods Mol. Biol. 526, 129–134. doi: 10.1007/978-1-59745-494-0_11 19378009

[B23] Shin H. Shin H.-S. Dewbre G. R. Harrison M. J. (2004). Phosphate transport in Arabidopsis: Pht1;1 and Pht1;4 play a major role in phosphate acquisition from both low- and high-phosphate environments. Plant J. 39, 629–642. doi: 10.1111/j.1365-313X.2004.02161.x 15272879

[B24] Vejchasarn P. Lynch J. P. Brown K. M. (2016). Genetic variability in phosphorus responses of rice root phenotypes. Rice 9. doi: 10.1186/s12284-016-0102-9 PMC4905936 27294384

[B25] Verma V. Srivastava A. K. Gough C. Campanaro A. Srivastava M. Morrell R.. (2021). SUMO enables substrate selectivity by mitogen-activated protein kinases to regulate immunity in plants. Proc. Natl. Acad. Sci. 118 (10), e2021351118. doi: 10.1073/pnas.2021351118 33649235 PMC7958252

[B26] Wang H. Sun R. Cao Y. Pei W. Sun Y. Zhou H.. (2015). OsSIZ1, a SUMO E3 ligase gene, is involved in the regulation of the responses to phosphate and nitrogen in rice. Plant Cell Physiol. 56, 2381–2395. doi: 10.1093/pcp/pcv162 26615033

[B27] Wissuwa M. Ae N. (2001). Further characterization of two QTLs that increase phosphorus uptake of rice (Oryza sativa L.) under phosphorus deficiency. Plant Soil 237, 275–286.

[B28] Xiujie Z. Wujun J. Wentao X. Xiaying L. Ying S. Sha L.. (2019). Comparison of five endogenous reference genes for specific PCR detection and quantification of rice. Rice Sci. 26 (4), 248–256. doi: 10.1016/j.rsci.2019.04.005

[B29] Zhang Y. Zeng L. (2020). “Crosstalk between ubiquitination and other post-translational protein modifications in plant immunity,” in Plant Communications (United Kingdom: Cell Press). doi: 10.1016/j.xplc.2020.100041 PMC7748009 33367245

[B30] Zhou J. Jiao F. Wu Z. Li Y. Wang X. He X.. (2008). OsPHR2 is involved in phosphate-starvation signaling and excessive phosphate accumulation in shoots of plants. Plant Physiol. 146 (4), 1673–1686. doi: 10.1104/pp.107.111443 18263782 PMC2287342

