# Corrigendum: SUMOylation of OsPSTOL1 is essential for regulating phosphate starvation responses in rice and *Arabidopsis*

**DOI:** 10.3389/fpls.2024.1412657

**Published:** 2024-06-04

**Authors:** Vaishnavi Mukkawar, Dipan Roy, Kawinnat Sue-ob, Andrew Jones, Cunjin Zhang, Prakash Kumar Bhagat, Sumesh M. Kakkunnath, Sigrid Heuer, Ari Sadanandom

**Affiliations:** ^1^ Department of Biosciences, Durham University, Durham, United Kingdom; ^2^ Department of Biochemistry, Cell and Systems Biology, Institute of System, Molecular and Integrative Biology, University of Liverpool, Liverpool, United Kingdom; ^3^ Department of Crop Science, Cambridge, Discovery LTD, Cambridge, United Kingdom

**Keywords:** post-translational modification, SUMOylation, phosphate-starvation tolerance 1 (OsPSTOL1), inorganic phosphate, phosphate deficiency

**Incorrect Funding**

In the published article, there was an error in the Funding statement. The wrong funder was listed. A correction has been made to the Funding statement:

The author(s) declare financial support was received for the research, authorship, and/or publication of this article. UKRI funding supported he PhB studentship.

The correct Funding statement appears below:

The author(s) declare financial support was received for the research, authorship, and/or publication of this article. This work was supported by The Biotechnology and Biological Sciences Research Council (BB/V003534/1).

The authors apologize for this error and state that this does not change the scientific conclusions of the article in any way. The original article has been updated.

