# Supplementary material for: SUMOylation of OsPSTOL1 is essential for regulating phosphate starvation responses in rice and Arabidopsis
Source: Front Plant Sci. 2024 Mar 7;15:1274610. doi: 10.3389/fpls.2024.1274610 (PMC10954814; doi:10.3389/fpls.2024.1274610)
Supplement: Supplementary file 1 [file DataSheet_1.pdf]

Supplementary material

Figure S1

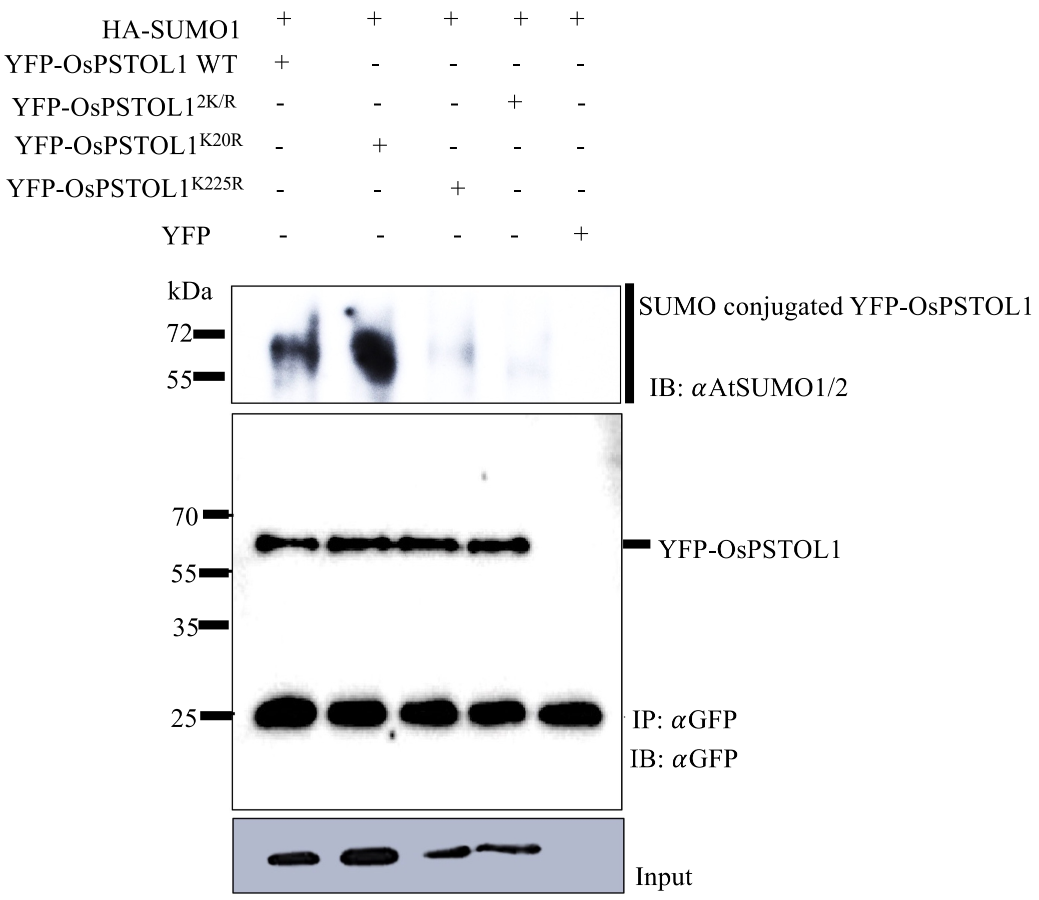

Figure S1: Immunoblot illustrating the SUMO conjugation of YFP-OsPSTOL1 WT, YFP- OsPSTOL1$^{2K/R}$, single mutants YFP-OsPSTOL1^{K20R} and YFP-OsPSTOL1^{K225R} in leaves of *N.benthamiana*

Immunoblot analysis of YFP-OsPSTOL1 WT, YFP- OsPSTOL1$^{2K/R}$, single mutants YFP-OsPSTOL1^{K20R} and YFP-OsPSTOL1^{K225R} proteins in *N. benthamiana* for SUMO conjugation. YFP-OsPSTOL1 WT, YFP- OsPSTOL1$^{2K/R}$, single mutants YFP-OsPSTOL1^{K20R} and YFP-OsPSTOL1^{K225R} constructs were transiently co-expressed with HA-SUMO in leaves of *N.benthamiana*. Immunoprecipitation (IP: αGFP) experiment was carried out with αGFP antibody beads from total protein extracted from leaves of *N.benthamiana*. Immunoblots were probed with αGFP (IB: αGFP) and αAtSUMO1/2 (IB: αAtSUMO1/2) antibodies respectively. SUMO-conjugated OsPSTOL1 is indicated by a black bar.

Figure S2A

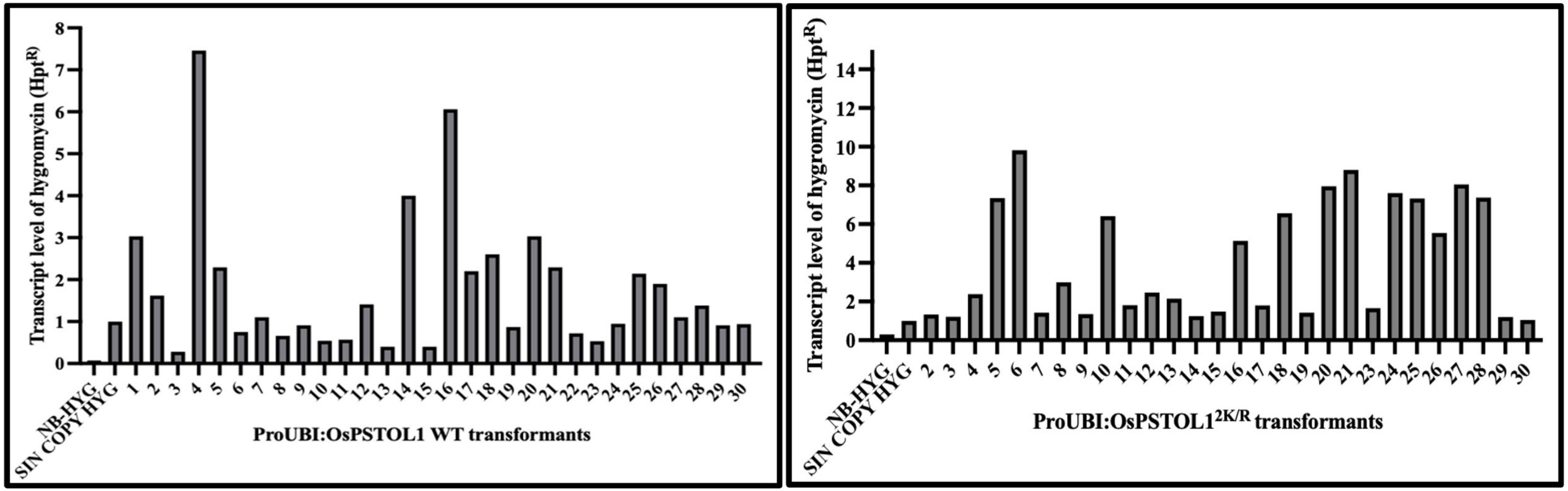

Figure S2A: Analysis of gene copy number in rice transgenics.
Pure genomic DNA was isolated from rice seedlings. Primers were designed to amplify the Hygromycin gene (HptR) and Sucrose Phosphate Synthase (SPS) gene to determine gene copy number. SIN COPY HYG is a positive control for hygromycin resistance gene in transgenic rice plants with single-copy gene insertion. Nipponbare (NB-HYG) was taken as a negative control with no insertion of the transgene.

Figure S2B

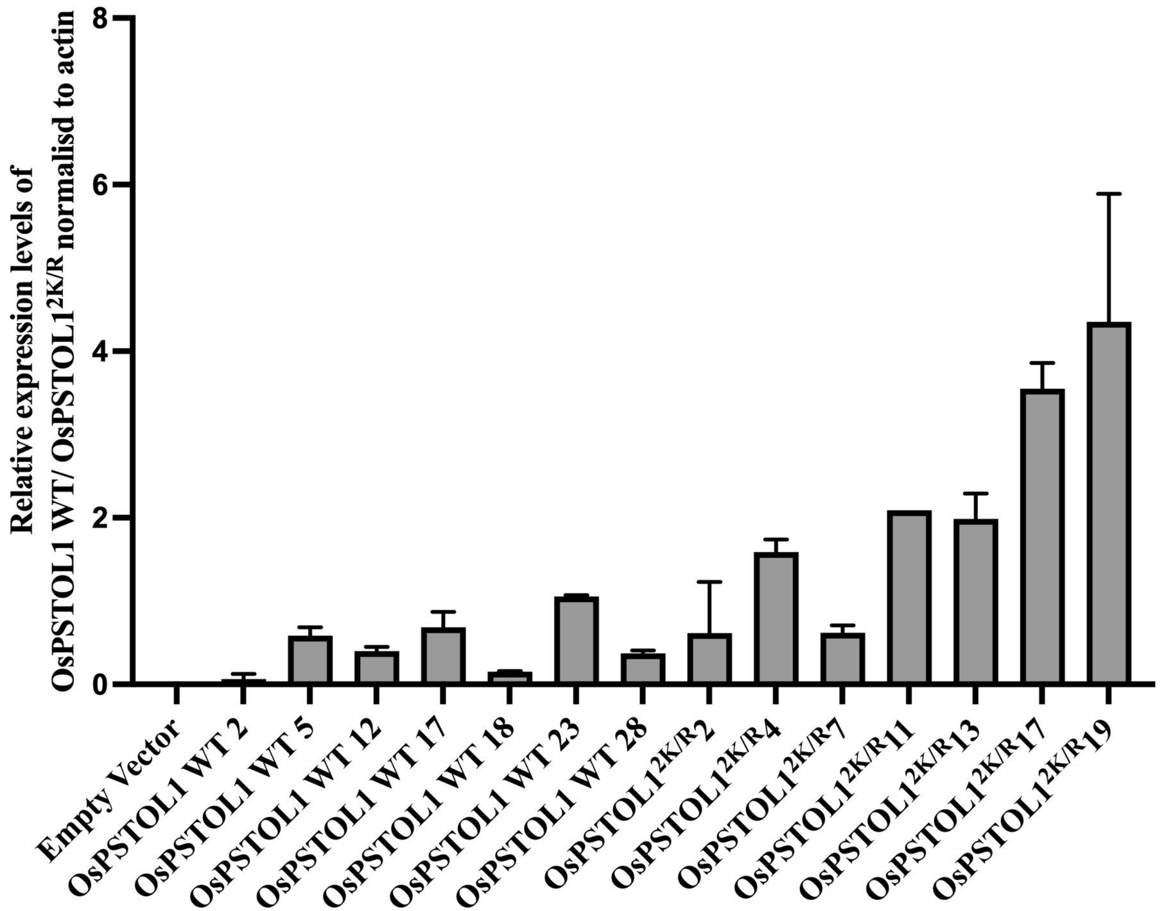

Figure S2B: Analysis of OsPSTOL1 transcript levels in YFP-OsPSTOL1 WT and YFP- OsPSTOL1$^{2K/R}$ rice transgenic lines.

Transcript analysis of OsPSTOL1 in empty vector (Nipponbare seedlings), OsPSTOL1 WT and OsPSTOL1$^{2K/R}$ rice transgenic lines. Total RNA was isolated from 10-day-old seedlings of all independent lines from each genotype and cDNA was prepared from the RNA by reverse transcription. The cDNA was diluted in a 1:5 ratio and resulted in cDNA being used as a template for qRT-PCR and the expression was normalised by actin which was used as an internal control. Error bars represent SEM.

Figure S3

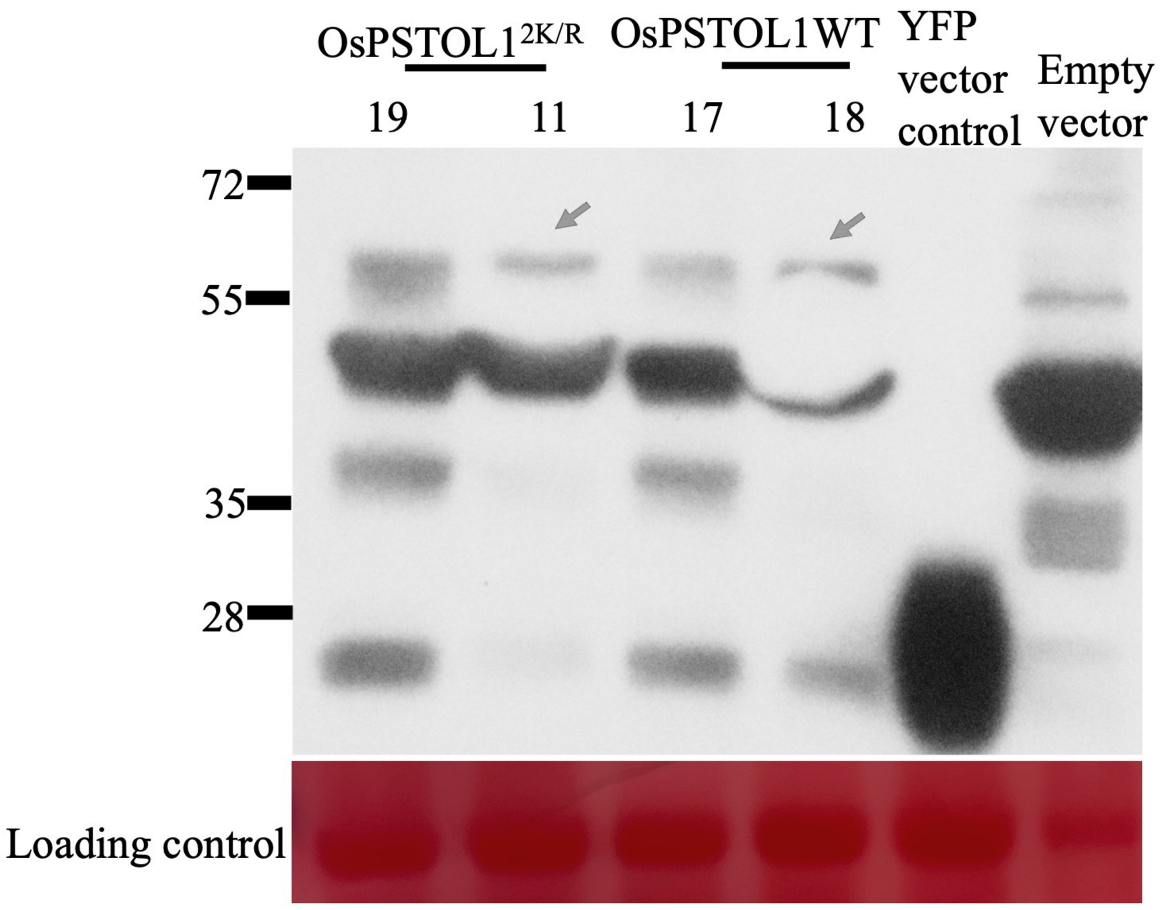

Figure S3: Western blot analysis showing target protein levels in each independent line of UBI::OsPSTOL1 WT and UBI::OsPSTOL1$^{2K/R}$ rice transgenic lines.
14 days old rice seedlings were used to extract total protein using 1x Laemmli sample buffer in a 1:1 ratio and subjected to SDS-PAGE analysis. The proteins were transferred to the PVDF membrane and immunoblot using αGFP antibodies. The protein band of PSTOL1 kinase was seen in both OsPSTOL1 WT and OsPSTOL1$^{2K/R}$ independent lines (shown by grey arrow) but the band corresponding to PSTOL1 kinase is absent from empty vector and YFP control. Nipponbare rice plant without PSTOL1 insertion (empty vector) was taken as a negative control. 30μl of total protein was loaded on SDS-PAGE.

Figure S4

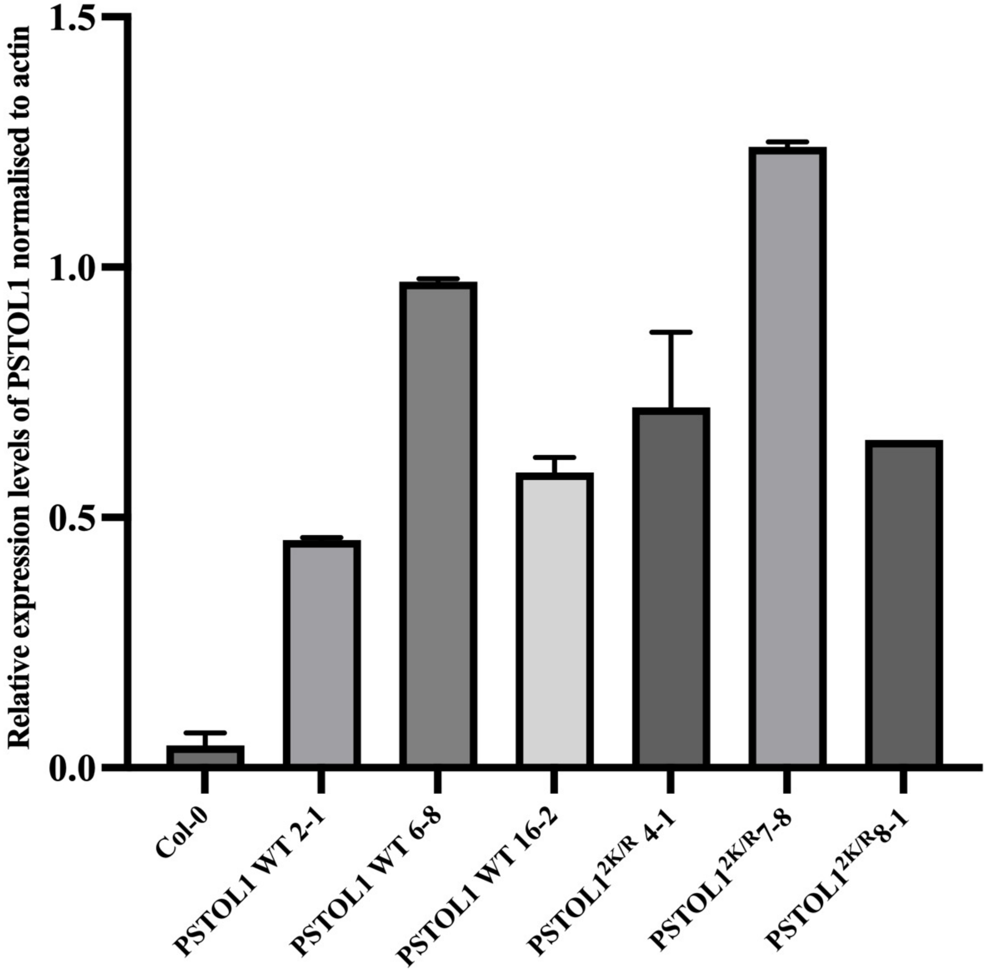

Figure S4: Analysis of Os*PSTOL1 WT/OsPSTOL1*$^{2K/R}$ gene expression.

Gene expression was analysed in 10 days old Arabidopsis seedlings relative to actin as a housekeeping gene. Total RNA was isolated from 10-day-old seedlings of all independent lines from each genotype and cDNA was prepared from the RNA by reverse transcription. The cDNA was diluted in a 1:5 ratio and resulted in cDNA being used as a template for qRT-PCR Error bars representing SEM of expression.

Figure S5

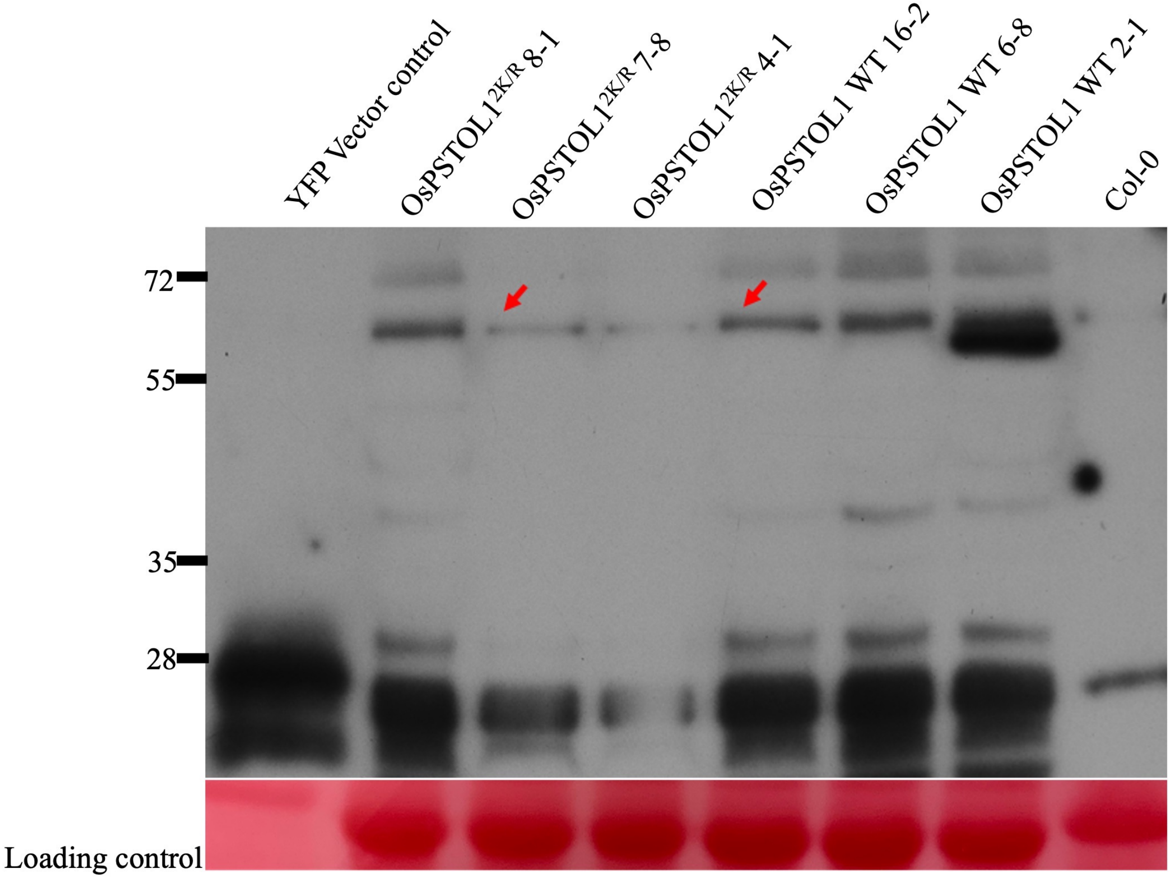

Figure S5: Western blot analysis showing protein levels in each independent line of 35S::PSTOL1 WT and 35:: PSTOL1$^{2K/R}$ transgenic Arabidopsis lines.
10 day-old Arabidopsis transgenic seedlings were used to extract total protein using 1x laemmli sample buffer in 1:1 ratio and subjected to SDS-PAGE analysis. The proteins were transferred to the PVDF membrane and immunoblotted using αGFP antibodies. The protein band of PSTOL1 kinase was seen in both PSTOL1 WT and PSTOL1$^{2K/R}$ independent lines (shown by red arrow) but the band corresponding to PSTOL1 kinase in absent from Col- 0 and YFP control. *Col-0* Arabidopsis seedlings without PSTOL1 insertion (empty vector) were utilised as a negative control. 30µl of total protein was loaded for SDS-PAGE analysis.

Supplementary Table S1: Primers used in this study

Gene Name	Forward sequence	Reverse sequence	Expected size (bp)
pMAL c5X_ OsPSTOL1	ACG CGT CGA CAT GGA TTA CAA GGA TGA CGA C	AAA CTG CAG TCA AAG CCC TTT TGG TGG	975
pMAL Vector specific primer	GGT CGT CAG ACT GTC GAT GAA GCC	TGT CCT ACT CAG GAG AGC GTT CAC	260
qPCR_ OsPSTOL1	CTG AGC TGGGAT AGA CTG TT	GGT GTTCCTCTTAGTCCGTT	216
qPCR_ OsActin	GACCCAGATCATGTTTGA GACCT	CAGTGTGGCTGACACCAT CAC	130
qPCR_ AtActin	CTTGCACCAAGCAGCATG A A	CCGATCCAGACACTGTAC T TCCTT	68
qPCR_ SPS	AGAGATCGACGAAAA	TTTTCGGGATGATCCGAG CC	104
qPCR_ Hygromycin	ACTGTCGGGCGTACA	GGTTTCCACTATCGG	85
